# Lattice disorder effect on magnetic ordering of iron arsenides

**DOI:** 10.1038/s41598-019-56301-5

**Published:** 2019-12-27

**Authors:** Athena S. Sefat, Xiaoping P. Wang, Yaohua Liu, Qiang Zou, Mimgming Fu, Zheng Gai, Kalaiselvan Ganesan, Yogesh Vohra, Li Li, David S. Parker

**Affiliations:** 1 Materials Science & Technology Division, Oak Ridge National Laboratory, Oak Ridge, TN 37831 USA; 2 Neutron Scattering Division, Oak Ridge National Laboratory, Oak Ridge, TN 37831 USA; 3 Center for Nanophase Materials Sciences, Oak Ridge National Laboratory, Oak Ridge, TN 37831 USA; 4 Department of Physics, University of Alabama at Birmingham, Birmingham, AL 35294 USA

**Keywords:** Materials science, Condensed-matter physics

## Abstract

This study investigates magnetic ordering temperature in nano- and mesoscale structural features in an iron arsenide. Although magnetic ground states in quantum materials can be theoretically predicted from known crystal structures and chemical compositions, the ordering temperature is harder to pinpoint due to potential local lattice variations that calculations may not account for. In this work we find surprisingly that a locally disordered material can exhibit a significantly larger Néel temperature (*T*_N_) than an ordered material of precisely the same chemical stoichiometry. Here, a EuFe_2_As_2_ crystal, which is a ‘122’ parent of iron arsenide superconductors, is found through synthesis to have ordering below *T*_N_ = 195 K (for the locally disordered crystal) or *T*_N_ = 175 K (for the ordered crystal). In the higher *T*_N_ crystals, there are shorter planar Fe-Fe bonds [2.7692(2) Å vs. 2.7745(3) Å], a randomized in-plane defect structure, and diffuse scattering along the [00 L] crystallographic direction that manifests as a rather broad specific heat peak. For the lower *T*_N_ crystals, the *a*-lattice parameter is larger and the in-plane microscopic structure shows defect ordering along the antiphase boundaries, giving a larger *T*_N_ and a higher superconducting temperature (*T*_c_) upon the application of pressure. First-principles calculations find a strong interaction between *c*-axis strain and interlayer magnetic coupling, but little impact of planar strain on the magnetic order. Neutron single-crystal diffraction shows that the low-temperature magnetic phase transition due to localized Eu moments is not lattice or disorder sensitive, unlike the higher-temperature Fe sublattice ordering. This study demonstrates a higher magnetic ordering point arising from local disorder in 122.

## Introduction

A bulk magnetic transition in a quantum material is thermally-driven by spin interactions dictated by nano- and mesoscale structures, such as lattice composition, crystal structure, disorder and defects, lattice strain, chemical impurities and dopants. The parents of the iron-based superconductors with *A*Fe_2_As_2_ (*A* = Ba, Sr, Ca, Eu) formula, known as ‘122’, are semi-metallic with all five Fe 3*d*-bands crossing the Fermi level^1^. These materials have a tetragonal structure at room temperature (*a* = *b* ≠ *c*), and at a Néel antiferromagnetic transition temperature (*T*_N_) there is a small tetragonal-to-orthorhombic structural distortion (*T*_s_) where the unit cell rotates by ~45° within the *ab*-plane^2,3^. Below *T*_N_ there is a sinusoidal modulation of Fe moments in the form of a spin-density wave (SDW)^3^ described by a wave vector ***q*** = (½ ½ 1) in the tetragonal structure, matching the nesting vector between the electron and hole pockets at the Fermi surface^4^. The Fe spin lattice is a ‘stripe’ arrangement (spins are antiparallel along *a-* and *c-*axes, and parallel along *b-*axis)^5–8^.

It has been found that strain can induce a nematic phase transition in 122 s^9^ that is seen as an electronic in-plane anisotropy^10^, driven by orbital (unequal occupation of *d*_xz_ and *d*_yz_) or spin directional order (not long range) that causes breaking of the in-plane C4 rotational symmetry and splits the *T*_N_ and *T*_s_ transitions. Moreover, higher *T*_N_ in BaFe_2_As_2_ is linked to a more homogenous electronic structure^11–14^, and there are local structure variations^3^. Thermally annealing of BaFe_2_As_2_ is found to shift and sharpen the heat capacity anomaly^15^. With disorder via electron irradiation in BaFe_2_(As_1−x_P_x_)_2_ the magneto-structural transition is suppressed and the superconducting dome tracks the shift of the antiferromagnetic phase^16^, while in Ba_1-x_K_x_Fe_2_As_2_ the antiferromagnetic and superconducting transition temperatures decrease^17^. For SrFe_2_As_2_, in addition to a large variability of *T*_N_ values (195 to 220 K)^6,13,18^, superconducting signature (filamentary *T*_c_ = 21 K) can be found in strained crystals^19^. Furthermore, CaFe_2_As_2_ can be synthesized as entirely non-magnetic, or an antiferromagnet with a large *T*_N_, achieved by staggered alleviation of local Fe−As bonds with thermal annealing^13,20,21^. For this study, we hypothesize that the local lattice details, including disorder, of EuFe_2_As_2_ may affect its *T*_N_ value. EuFe_2_As_2_ is unique among the 122 s for having both SDW order of Fe, and Eu local moment order below ~20 K with ***q*** = (0 0 L)^22–25^. The Eu local moments are weakly coupled to the Fe sublattice and are strong enough for a large and indirect spin-lattice coupling that can lead to a structural detwinning by a small magnetic field^26^ and can cause re-entrant non-bulk superconductivity^27,28^.

We have synthesized two stoichiometric EuFe_2_As_2_ crystals with onset of Fe ordering at *T*_N_ = 195 K or *T*_N_ = 175 K, and use X-ray and neutron diffraction, microscopy and spectroscopy techniques, and theory, to understand the reasons that give these magnetic ordering temperatures. Surprisingly, the higher *T*_N_ crystal has a broader (usually associated with strain and disorder) specific heat peak, while the lower *T*_N_ crystal has a sharp peak. How does *T*_N_ relate to lattice and topological features, and does *T*_N_ value correlate with averaged lattice parameters? Would higher *T*_N_ mean more homogeneous electronic structure? What are the pressure results for diminishing antiferromagnetism and potentially deriving superconductivity in each of these crystals? Our results show that although *T*_N(Fe)_ and *T*_c_ values are greatly sensitive to the lattice details and local arrangements of defect structures, Eu ordering is unaffected at *T*_N(Eu)_ ≈ 20 K. This study demonstrates the apparent contradictory result that certain types of disorder can significantly increase the magnetic order, even in stoichiometric quantum material.

## Experimental Procedure and Results

Single crystals of EuFe_2_As_2_ were grown out of a mixture of Eu and FeAs excess used as liquid flux^12,29,30^. Each of these mixtures was warmed to 1180 °C, then cooled (2 °C*/*h) followed by a decanting of the FeAs excess flux at 1090 °C. Two different reaction loading ratios were used to obtain EuFe_2_As_2_ single crystals with different *T*_N_. A loading ratio of Eu:FeAs = 1:5 gives an onset transition temperature of *T*_N_ = 195 K (referred to as ‘crystal a’), while Eu:FeAs = 1:4 gives crystals with *T*_N_ = 175 K (‘crystal b’). The chemical composition of these crystals was measured with a Hitachi S3400 scanning electron microscope operating at 20 kV. Three spots (each ∼80 μm area) were checked and averaged on each crystal; energy-dispersive X-ray spectroscopy (EDS) and site-refinement of single-crystal X-ray diffraction analyses indicate that both crystals are stoichiometric and are EuFe_2_As_2_. The phase purity of the crystals was checked by collecting data on an X’Pert PRO MPD X-ray powder diffractometer; structures were solely identified as tetragonal ThCr_2_Si_2_ structure type (*I*4*/mmm*, *Z* = 2).

**Figure 1:**
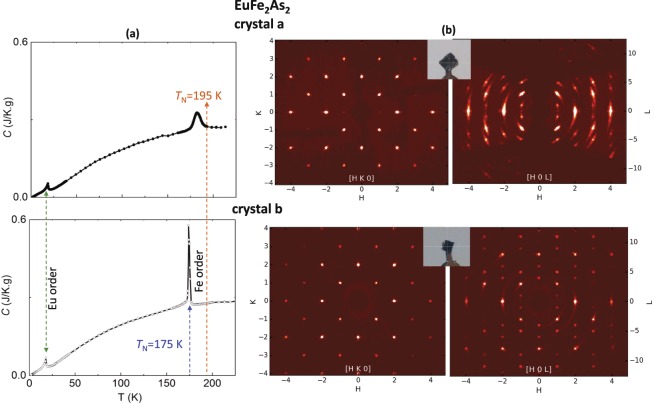
Data on EuFe_2_As_2_ ‘crystal a’ and ‘crystal b’. (**a**) Specific heat, C(T), results with transitions associated with Eu (*T*_N_ = 21 K) and Fe ordering (*T*_N_ = 195 K or 175 K). (**b**) Precession images showing the [HK0] and [H0L] reciprocal lattice planes reconstructed from single crystal X-ray diffraction patterns measured at room temperature. Smearing in diffraction spots are more prevalent in ‘crystal a,’ noted in [H0L] plane.

Specific heat data were collected on EuFe_2_As_2_ single crystals, using a Quantum Design Physical Property Measurement System (PPMS); the C(T) results are shown in Fig. 1a. Each EuFe_2_As_2_ crystal exhibits two transitions: an ordering of the Fe lattice at higher temperature, followed by lower temperature ordering (≈20 K) due to Eu moments. The EuFe_2_As_2_ ‘crystal a’ exhibits the higher onset ordering temperature of *T*_N_ = 195 K with a broader peak, compared to ‘crystal b’ giving a sharp lambda transition below *T*_N_ = 175 K. The specific heat result of ‘crystal a’ looks similar to that reported in a pressed polycrystalline sample (with respect to peak width, height, and transition temperature)^22^.

To analyze crystal structures, single crystal X-ray diffraction data were collected at room temperature on a Rigaku XtaLAB PRO diffractometer (Mo Source, K_α_ = 0.71073 Å) equipped with a DECTRIS Pilatus 200 K area detector. Data collection and reduction used the CrysAlisPro program^31^. Crystal structure refinements were performed using the SHELX-2014 program^32^. Figure 1b (insets) shows the pictures of crystals for the data collection, stuck on top of needles. Crystals ‘a’ and ‘b’ appeared similar visually, with sheet morphologies; [001] direction is perpendicular to the plane of the plate. Figure 1b gives the precession images of [HK0] and [H0L] reciprocal lattice planes, showing lattice strain effects associated with smeared diffraction spots in the L direction for ‘crystal a’; this crystal has a relatively broad C(T) peak (*T*_N_ = 195 K) from the Fe ordering. A broader peak of specific heat is usually associated with lattice strain and disorder. Table 1 shows the lattice parameters and refinement results of the X-ray structure measured at room temperature. The EuFe_2_As_2_ sample with higher *T*_N_ (‘crystal a’) gives a lower overall *a*-lattice parameter and a shorter planar Fe−Fe distance of 2.7692(2) Å, indicating a contraction of 0.0053(4) Å for the in-plane Fe−Fe bond when compared to that of 2.7745(3) Å for the lower *T*_N_ (‘crystal b’).

**Table 1:** Single-crystal X-ray diffraction refinement on the two crystals of EuFe_2_As_2_: ‘crystal a’ (*T*_N_ = 195 K), ‘crystal b’ (*T*_N_ = 175 K).

EuFe_2_As_2_ sample ID	‘crystal a’	‘crystal b’
*a* (Å)	3.9162 (3)	3.9238 (4)
*c* (Å)	12.104 (3)	12.105 (2)
**As at (0, 0**, ***z*); 4e**		
*z*	0.36256 (13)	0.36255 (11)
*U*_iso_	0.0104 (6)	0.0109 (6)
arsenic height, Å	1.3624 (10)	1.3624 (8)
site occupancy	0.96 (9)	0.97 (8)
**Fe at (½, 0, ¼); 4*d***		
*U*_iso_	0.0106 (6)	0.0107 (6)
Fe‒Fe distance, Å	2.7692 (2)	2.7745 (3)
site occupancy	0.97 (9)	0.97 (8)

**Figure 2:**
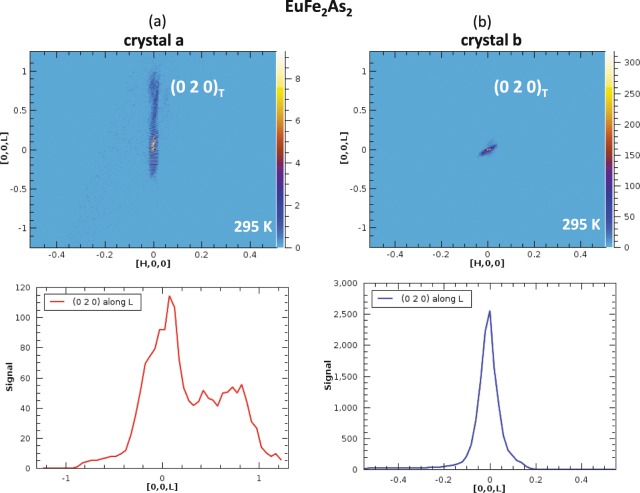
Tetragonal (0 2 0) peak profiles for the two EuFe_2_As_2_ crystals at room temperature for (**a**) ‘crystal a’ and (**b**) ‘crystal b’, showing the peak false color maps, and their corresponding peak intensity profiles along the crystallographic *c* direction. Note the difference in scales on both axes. The peak from ‘crystal a’ is substantially broadened showing extended diffuse lines.

Neutron diffraction measurements were carried out using the TOPAZ single crystal diffractometer at the ORNL Spallation Neutron Source (SNS), which uses the wavelength-resolved Laue technique with an extensive array of neutron time-of-flight area detectors for 3-dimentional *Q* space mapping of Bragg and diffuse scattering patterns originating from magnetic and nuclear phase transitions^33^. Pieces of ‘crystal a’ and ‘crystal b’ had dimensions of 3.25 × 2.50 × 0.20 mm^3^ and 2.75 × 2.63 × 0.30 mm^3^, respectively, and were attached on a MiTegen loop using super glue for data collection at room temperature and 95 K. Each of the samples was oriented with high precision for volumetric sampling of Bragg peaks in specific directions with the CrystalPlan program^34,35^. At room temperature, the (0 2 0)_Tetragonal_ peak profiles for the two EuFe_2_As_2_ crystals are shown in Fig. 2, demonstrating the peak false-color maps and their corresponding peak intensity profiles along the crystallographic *c*-direction. The peak from ‘crystal a’ is broad and shows extensive diffuse lines along the L direction, which is consistent with that observed in X-ray diffraction, and gives broader and higher *T*_N_ in C(T).

**Figure 3:**
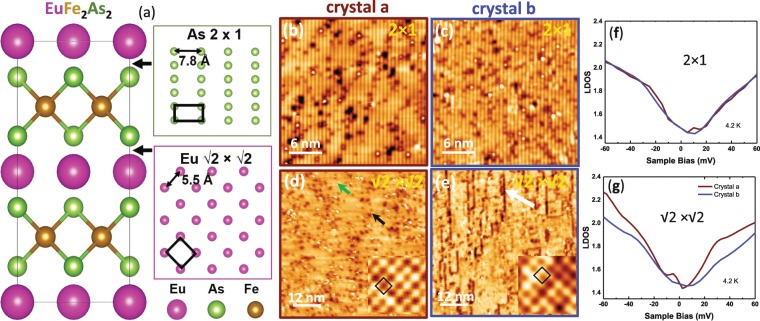
(**a**) The structure model of EuFe_2_As_2_ and the surface reconstruction models of arsenic 2 × 1 and europium √2 × √2 terminations. The black boxes outline the unit cells. (**b**,**c**) Topographic images of 2 × 1 surface reconstruction from ‘crystals a’ (−500 mV, 100 pA) and ‘crystal b’ (−1 V, 100 pA), respectively. (**d**,**e**) Topographic images of √2 × √2 surface reconstruction from ‘crystals a’ (−60 mV, 400 pA) and ‘crystal b’ (−20 mV, 800 pA), respectively. Insets show the atomic resolved images in 3 × 3 nm^2^ size and black boxes are the unit cells of √2 × √2 surface reconstruction. (**f**,**g**) Comparison of average LDOS from ‘crystal a’ (red) and ‘crystal b’ (blue), for surface reconstructions at 4.2 K.

As shown in Fig. 1b, the streaks for the (2 0 0) peak along L direction for ‘crystal a’ is much more pronounced (Fig. 2a) than that of ‘crystal b’ (Fig. 2b), which could be induced by strain, stacking fault or disorder in the bulk single crystal sample. Since the refined average *c* lattice parameter remains essentially unchanged for both crystals (Table 1), the much more pronounced streaks for ‘crystal a’ are likely from microstrain caused by randomly distributed defects in ‘crystal a’, which is evident from scanning tunneling microscopy/spectroscopy (STM/S) measurement (see Fig. 3 below). The split of the peaks along *c* also indicates twinning and multidomain structures of ‘crystal a’. In particular, we note from Fig. 2 (bottom panels) that the full-width at half maximum (FWHM) for the (2 0 0) peak along L direction is some 0.13–0.27 reciprocal lattice units larger than that of ‘crystal b.’ If one assumes that in the ordered ‘crystal b’ the finite (i.e. non-zero) FWHM is largely due to measurement resolution limits, the FWHM for ‘crystal a’ would correspond to a ~2–5% local variation in the lattice constant *c*.

For these crystals, neutron diffraction results were additionally carried out at 95 K, to confirm the Fe magnetic peak below *T*_*N*_ in accordance with the propagation vector ***q*** = (1 0 1)_Orthorhombic_ = (½ ½ 1)_Tetragonal_ and the stripe arrangement of the Fe spin lattice^5–8^ (see Fig. S1a in Supplementary). Additionally, variable temperature scans of the Eu magnetic peak at (0 3 0) for both crystals were performed below 30 K using the elastic diffuse scattering spectrometer CORELLI at SNS^32^ and showed the same temperature dependence of Eu magnetic peak as (0 3 0) with *T*_N_ = 21 K (see Fig. S1b). In contrast, the Néel transition temperature for Fe is clearly lattice and disorder sensitive. The high-temperature phase transition is due to the ordering of the transition metal Fe moments, whereas the low-temperature phase transition is due to the ordering of the localized Eu moments^22^. The divergent behavior of the Fe and Eu sublattices from ‘crystal a’ to ‘b’ – the former sublattice sensitive to lattice disorder, the latter insensitive – is of interest here. One plausible hypothesis is that the Eu 4 *f* moments are much more localized, near the Eu nucleus, than the Fe moments that are more itinerant. This would suggest that the Eu 4 *f* moments are less subject to nano-strain than the Fe 3*d* moments, and the relatively isolated location of Eu between the layers (as opposed to the tightly bound FeAs layers) would enhance this possibility. Supporting this assertion is the relative insensitivity of Eu ordering temperature to pressure (see Fig. S4), again unlike the Fe sublattice ordering. Indeed the Eu magnetic order survives to higher pressure than the Fe order, despite occurring at much lower temperatures.

To further investigate the local origin of the bulk *T*_N_ differences between the two EuFe_2_As_2_ crystals, surface topography and electronic structures were investigated using STM/S on *in-situ* low-temperature cleaved surfaces. The two sets of EuFe_2_As_2_ ‘crystal a’ and ‘crystal b’ were cleaved in an ultra-high vacuum at ~ 78 K and then immediately transferred to the STM/S head precooled to 4.2 K without breaking vacuum. The STM/S experiments were carried out using a scanning tunneling microscope with base pressure better than 2 × 10^−10^ Torr, with mechanically cut Pt-Ir tip. All Pt-Ir tips were conditioned on clean Au (1 1 1) and checked using the topography, surface state, and work function before each measurement. The STM/S were controlled by the SPECS Nanonis control system. Topographic images were acquired in constant current mode with bias voltage applied to samples, and tip grounded. All the spectroscopies were obtained using the lock-in technique with a modulation of 1 mV at 973 Hz on bias voltage, dI/dV. Current-imaging-tunneling-spectroscopy were collected over a grid of pixels at bias ranges around Fermi level using the same lock-in amplifier parameters. The survey on multiple large areas of both samples shows the coexistence surface reconstructions on ‘crystal a’ and ‘crystal b’, as shown in Fig. 3. Using a similar method as STM report on Co-doped BaFe_2_As_2_^36^, the 2 × 1 and √2 × √2 reconstructed surfaces can be assigned to arsenic termination and europium termination, respectively, as shown in Fig. 3a. While the 2 × 1 surfaces of both crystals (Fig. 3b,c) are very similar, the √2 × √2 surfaces of the two crystals (Fig. 3d,e) are very different. The atomic resolved images in the insets of Fig. 3d,e from the well-ordered √2 × √2 reconstructed areas of the two crystals are similar, but the arrangements of the defects on the surfaces are rather different. In ‘crystal a’, surface defects (one of the defects is marked with a black arrow in Fig. 3d) are essentially randomly distributed on the surface, but in ‘crystal b’, large amount of defects prefer to form into chains (one of the chains is marked with a white arrow in Fig. 3e). By analyzing atomically resolved images around these areas, we found the chains are aligned on the antiphase boundaries. Because the surface reconstruction is 2 × 1 or √2 × √2, those domains can shift by 1 to form antiphase boundaries. Although antiphase boundaries also exist on the ‘crystal a’ surface (one of the antiphase boundaries is marked with a green arrow in Fig. 3d), defects in ‘crystal a’ do not segregate along the boundaries. The electronic properties revealed by the STS from the surfaces are consistent with the morphological observation (see also Fig. S2). In Fig. 3f, the average electronic local density of states (LDOS) of 2 × 1 surfaces (As termination) over large areas are almost identical for both crystals, but the LDOS from the two √2 × √2 surfaces (Eu termination) in Fig. 3g have significant differences. This shows that the different arrangements of the surface defects in the two crystals change the electronic structure dramatically. Given the preparation of these surfaces by cleaving the crystals in ultra-high vacuum at low temperatures, this difference is in electronic structure that is generally reflective of the effects of the defects in the bulk crystals.

For both EuFe_2_As_2_ crystals, high-pressure electrical-resistance measurements were performed using a diamond anvil cell (see Figs. S3 and S4), to explore the differences of pressure effects on the two crystals that have different *T*_N_, under the same experimental setup. Although the feature due to Eu ordering is not changed for either crystals up to ~4 GPa, Fe ordering is greatly sensitive to pressure and the rate of *T*_N_ suppression for both crystals is similar. For ‘crystal b’ with smaller *T*_N_ = 175 K, the drop in resistivity is noticed at lower pressure of 2.5 GPa, compared to ‘crystal a,’ with a drop in resistivity at 3.2 GPa. EuFe_2_As_2_ with sharper, but lower *T*_N_, is prone to a higher superconducting dome (**S4**); highest *T*_c_ value for ‘crystal a’ is 36 K, and for ‘crystal b’ is 41 K.

## First Principles Calculations

In the effort to understand the sample-to-sample variation in Néel temperatures, we have conducted first principles theory calculations of the effect of nano-scale strain on the magnetic order, specifically on two questions. The first question is straightforward: is the observed variation in *T*_N_ related to the slight difference in planar lattice constants in crystals ‘a’ and ‘b’? We assess based on calculations of the ‘ordering energy,’ specifically the difference in energy between the stripe ground-state and the checkerboard (all neighboring moments are anti-aligned) excited state. The second question, with more complex interpretation, is whether *c*-axis strain is relevant to the observed *T*_N_ variation. The motivation for studying this question is the evident ‘shoulder’ in the neutron diffraction results presented in Fig. 2a, which could result from *c*-axis nano-scale strain, and thereby an effective *distribution* of local *c*-axis lattice parameters. Note that the bulk lattice constants for crystals ‘a’ and ‘b’ are identical (to within 0.001 Å), so that we are studying, in effect, the local nanoscale energetics rather than constructing an involved model of the bulk Néel point variation for a sample with a distribution of local lattice constants.

First principles calculations were performed using the linearized augmented plane wave (LAPW) density functional theory code WIEN2K^37^. As in our previous work^14^ we have used the local density approximation (LDA), with arsenic height and lattice parameters taken directly from our experimental XRD refinements (Table 1), except as stated below. An RK_max_ value of 8.0 – the product of the smallest sphere radius and largest planewave expansion wavevector – was used. To avoid the generally confounding complexities of 4 *f* physics (here we are focused on the Fe magnetic behavior) we have followed our previous theoretical work^38^ and performed the all-electron calculations with the isoelectronic substitution of Sr for the divalent Eu, but retained our lattice parameters and internal coordinates for EuFe_2_As_2_. The effect of these choices is to allow a direct evaluation of the Fe sublattice magnetic properties without the need to apply a correlated (i.e. LDA + U) approach to deal with the Eu 4 *f* electrons, which play little or no role in the Fe magnetism. Sphere radii of 2.5 Bohr were used for Sr, and between 2.26 and 2.30 for Fe (depending on volume), and between 2.15 and 2.22 for As. Identical sphere radii were used for the calculations at given volume, relevant for assessing the interlayer coupling.

With regards to the first question of whether changes in the magnetic ordering energy, or energetic difference between the stripe ground state and checkerboard excited state, result from the small difference in planar lattice parameters, we find only a small change (0.4%) in this energy, from 51.6 meV/Fe for ‘crystal a’, to 51.8 meV/Fe for ‘crystal b’. This means that the observed ~ 10% Néel temperature variation does not result from changes in this ordering energy. At the same time, one should note that the observed 0.2% change in lattice parameter from ‘crystal a’ to ‘b’ corresponds to an effective chemical pressure of several tenths of a GPA, and pressure is a well-known means of *T*_N_ suppression. While it remains possible that this effective planar strain is relevant here, arguing against this is the fact that the smaller lattice parameter ‘crystal a’ has the larger Néel point, whereas the application of pressure, thereby yielding smaller lattice constants, generally lowers the *T*_N_.

Concerning the second question, the neutron scan of ‘crystal a’ along the [00 L] direction shows clear evidence of disorder, with a rather broadened peak around the (0 0 0) point and a substantial ‘shoulder’ feature extending nearly to the [001] point (Fig. 2a). While there are many possible sources of such disorder, ranging from vacancies and anti-site defects to small grain sizes, here we explore the idea that *c*-axis strain in ‘crystal a’ is ultimately responsible for the rather broad neutron diffraction peaks. The hypothesis here is that nanoscale *c*-axis strain could significantly change the interlayer magnetic coupling, since this coupling is known from previous work^39^ to depend rather substantially on pressure, or equivalently compression or expansion of the cell from the equilibrium value. The interlayer coupling is of course necessary to attain a finite ordering temperature, given the Mermin-Wagner finding that truly low-dimensional systems do not order at finite temperature. Since, as in many materials, the interlayer coupling here is relatively weak, it is plausible that nanoscale *c*-axis strain could change the coupling, which generally falls off quickly with increased distance.

We have therefore conducted calculations of this interlayer magnetic coupling. Here we report the energy difference, per unit cell, between the ground-state stripe magnetic order (which is antiferromagnetically coupled along the *c*-axis) and a state with ferromagnetic *c*-axis coupling. We have done this at the experimental lattice parameter of 12.104 Å, and in addition for *c* values 5% larger and smaller than this value. For the experimental *c*-lattice parameter, we find an interlayer coupling energy difference of 7.1 mRyd/u.c., which increases slightly to 7.7 mRyd for the +5% *c* value, but decreases sharply to some 4.5 mRyd for the −5% *c* value. These values are in qualitative agreement with the previous work on BaFe_2_As_2_^39^, where it was found that the application of pressure decreases the interlayer coupling. We mention also that the calculated magnetic moments decrease substantially for the smaller cell, and correspondingly increase for the larger cell. In the antiferromagnetically coupled layer state, for the smallest cell these moments are 1.25 μ_B_ per Fe, for the experimental cell the value is 1.63 μ_B_, and for the largest cell is 1.92 μ_B_. Neutron diffraction results give an Fe moment of ~1 μ_B_^8^, consistent with the general overstatement of magnetic order by first principles approaches in these materials.

These data suggest that interlayer strain can affect the magnetism in a substantial manner. In this scenario, the broadened specific heat peak in ‘crystal a’ would result from a *distribution* of local lattice parameters, and thereby a distribution of local interlayer exchange couplings, resulting in a spread of ordering points. However, one difficulty with this argument is that such a distribution would have a larger effect on the temperature width of the transition than the mean transition temperature, since the bulk *c*-axis lattice parameters in crystals ‘a’ and ‘b’ are identical (leaving aside such questions as the temperature dependence of the magnetic correlation length in such a disordered scenario). Furthermore the fact that the strained calculations show larger *decreases* in magnetic order (measured as the interlayer coupling) with compressive strain than *increases* with tensile strain suggests that the effect of a distribution of *c*-axis lattice parameters around the bulk value would be a *decrease* in Néel point, not an increase. If the reverse were true – that the increases in magnetic order with one form of strain were larger than the decreases with the opposite strain – one would in fact have a possible explanation for the *T*_N_ enhancement. However, the calculations do not suggest this ladder possibility, and thus we must consider, as unsupported the hypothesis, that strain-related interlayer-magnetic exchange differences create the observed *T*_N_ increase in ‘crystal a’.

Since the straightforward explanations of the *T*_N_ increase, based on disorder-induced structure modification, do not explain this rather unusual behavior, it is necessary to consider other possibilities. For example, in materials near a magnetic instability, it has recently been posited^40^ that charge doping can induce a magnetic transition based on Stoner physics, and one could plausibly imagine a similar enhancement in magnetic character of an already magnetic material. However, the generally stoichiometric character of both samples studied here argues against such a possibility. An additional possibility is provided by the theoretical work of Gastiasoro *et al*.^41^, which finds (for superconductivity, and extended to magnetic order) density-of-states increase from either resonant states or Anderson localization leading to enhancements in ordering points. We here suggest a different possibility: spin fluctuations are known to be exceptionally strong in the iron arsenides, and in fact are a leading candidate for the interaction causing superconductivity. It is commonly believed^42^ that these fluctuations also play an important role in reducing the Néel point of these materials from a much higher ‘bare’ value. It is also known from several recent theory works^43,44^ that disorder can play a substantial role in weakening spin fluctuations and inducing magnetism, and this role is especially prominent^45^ in the case of stripe magnetic order, as is observed in the iron-arsenides. Putting such arguments together, we suggest that the disorder evident in our specific heat and neutron data is weakening the spin fluctuations and thereby enhancing the magnetic order.

One means of testing this theory would be to employ the quasi-particle interference techniques recently applied to LiFeAs^46^. While in that work it was the superconductivity (and its coupling to a bosonic excitation) that was studied, we anticipate that a measure suitably adapted to the case of the antiferromagnetic order here could also yield insight. If reduced spin fluctuations are in fact at work here, the momentum and energy dependent differential conductance g(**k**, ω) should show signatures of reduced scattering in the higher *T*_N_. In particular, generally sharper (as a function of momentum and energy) peaks are expected in the conductance in this material, when measured at the same temperature as the lower Néel point crystal. Future experimental studies would be highly beneficial are to assess this possibility.

## Conclusion

We find that disorder-related lattice variability drives significantly different Fe Néel ordering temperatures in the stoichiometric EuFe_2_As_2_ crystals, with the highly unusual result that the *more* locally disordered crystal exhibits the *higher* Néel point. The diffuse scattering that is seen along the [00 L] direction in X-ray and neutron diffraction measurements for the higher *T*_N_ crystal may well be the origin of the broadened specific heat peak and the suppressed superconducting dome. The surface topography and electronic structure studies show that there is a clear difference in electronic and defect structures between the two *T*_N_ crystals, with defect states dominating and elevating the LDOS for the higher *T*_N_ ‘crystal a’. Although the two crystals have a similar number of defects, their segregation around the antiphase boundaries in ‘crystal b’ largely decreases the number of random individual defects, which may cause a higher-*T*_*c*_ superconductor with pressure. This study thereby demonstrates the modification of *T*_N_ in an iron arsenide by controlled disorder, thus explaining the observation of substantial ordering temperature variations for these stoichiometric quantum materials in the literature.

## Supplementary information

Supplementary Information
