# Supplementary material for: Lattice disorder effect on magnetic ordering of iron arsenides
Source: Sci Rep. 2019 Dec 27;9:20147. doi: 10.1038/s41598-019-56301-5 (PMC6934717; doi:10.1038/s41598-019-56301-5)
Supplement: Supplementary file 1 — Supplementary Information [file 41598_2019_56301_MOESM1_ESM.docx]

**Supplementary Material**

for

**Lattice disorder effect on magnetic ordering of iron arsenides**

Athena S. Sefat,^1*^ Xiaoping P. Wang,^2^ Yaohua Liu,^2^ Qiang Zou,^3^ Mimgming Fu,^3^ Zheng Gai,^3^

Kalaiselvan Ganesan,^4^ Yogesh Vohra,^4^ Li Li,^1^ David S. Parker^1^

*^1^ Materials Science & Technology Division, Oak Ridge National Laboratory, Oak Ridge, TN 37831*

*^2^ Neutron Scattering Division, Oak Ridge National Laboratory, Oak Ridge, TN 37831*

^3^ *Center for Nanophase Materials Sciences, Oak Ridge National Laboratory, Oak Ridge, TN 37831, USA*

*^4^ Department of Physics, University of Alabama at Birmingham, Birmingham, AL 35294*


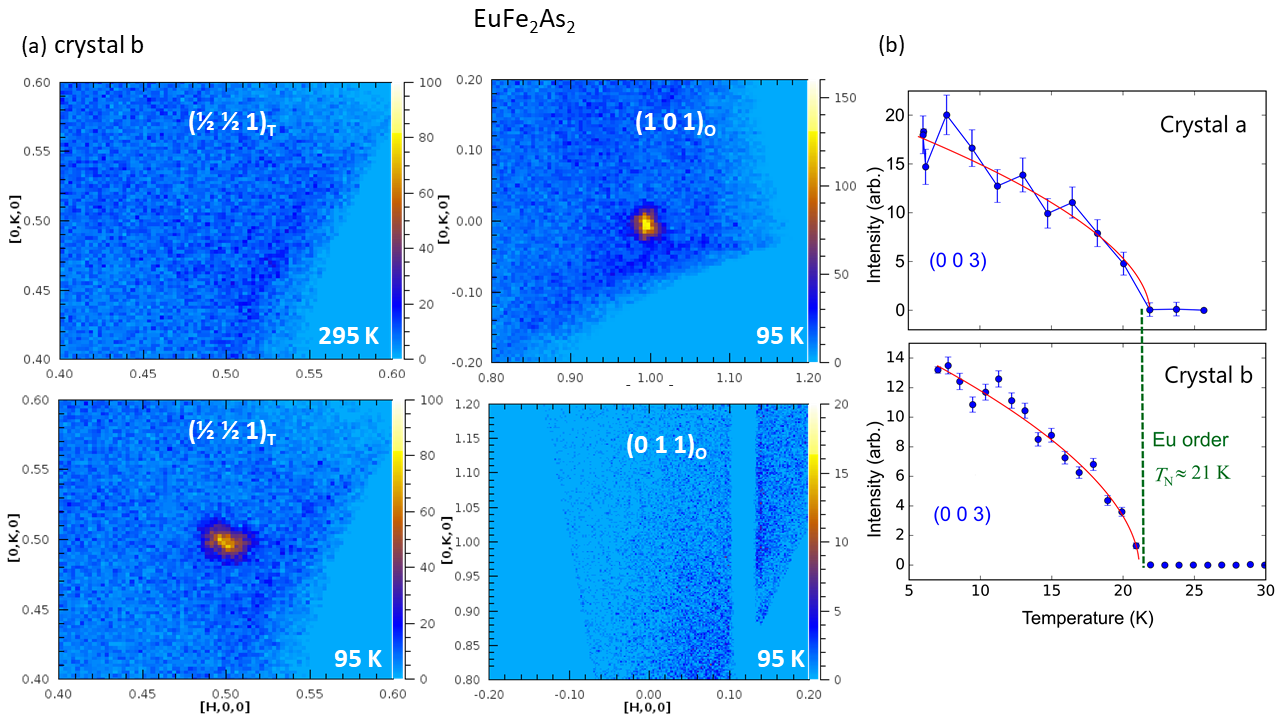


**Fig. S1**: For EuFe_2_As_2_: (a) Neutron single crystal diffraction showing the appearance of Fe magnetic peak for ‘crystal b’ below *T*_N_ in accordance with the propagation vector (1 0 1)_O_=(½ ½ 1)_T_. Left: There is no Fe magnetic peak in the tetragonal phase at room temperature, while Fe magnetic superlattice peak is seen at 95 K indexed in (½ ½ 1)_T_. Right: The same Fe magnetic peak indexed as (1 0 1)_O_ in the low temperature orthorhombic phase after basis transformation (***a***+***b***, -***b***+***a***, ***c***), while absence of (0 1 1)_O_ is consistent with ‘stripe’ arrangement of Fe spin lattice known for EuFe_2_As_2_ [1]. The Fe spins are antiparallel along *a*- and *c*-axes, and parallel along *b*-axis. No twinned domains are observed for ‘crystal b’. (b) Temperature dependence of the Eu (0 0 3) magnetic reflection for crystals ‘a’ and ‘b’. Both samples exhibit approximately the same ordering behavior in terms of ordering temperature (≈21 K) and critical exponents (0.28).

For EuFe_2_As_2_ crystals, neutron diffraction results were carried out at 95 K using TOPAZ single-crystal diffractometer, with the temperature controlled by an Oxford CryoStream with *l*-N_2_ flow. The appearance of an Fe magnetic peak below *T_N_* is confirmed in accordance with the propagation vector ***q***=(1 0 1)_O_=(½ ½ 1)_T_. Additionally, the absence of (0 1 1)_O_ reflection at temperatures below *T*_N_ are consistent with the assignment of Fe spins are antiparallel along *a-* and *c-*axes, and parallel along *b-*axis of the orthorhombic cell; these results are shown for ‘crystal b’ in **Fig. S1a**. The same EuFe_2_As_2_ single crystal samples ‘a’ and ‘b’ used for the TOPAZ experiments were remounted using an aluminum pin on the CORELLI cryostat for data collection below 30 K. Peak intensities for individual temperature scans used ~ 7 minutes of neutron beam time, with 0.5 Columbus of proton-charge on target as the stopping criteria for data collection at each temperature. The temperature dependent Eu magnetic peak of (0 3 0) shows approximately the same ordering for both crystals, with critical exponents 0.28(5) for ‘crystal a’ and 0.28(3) for ‘crystal b.’ These results are shown in **Fig. S1b**.

To better understand how the local chemical structure changes the electronic structure on √2 × √2 surfaces, the spatial distributions of LDOS at ± 60 mV from the two EuFe_2_As_2_ crystals are plotted in **Fig. S2** along with the simultaneously acquired topographic images. For ‘crystal a’, the intensities of the LDOS maps (**Fig. S2b**, **c**) are relatively higher and more uniform compared to ‘crystal b’ (**Fig. S2e**, **f**). What marks the difference between the two crystals are the large quantities of low density-of-state areas in ‘crystal b’ (green areas in **Fig. S2e** and dark blue areas in **Fig. S2f**), those are areas with fewer vacancy defects in the topographic images. It is interesting to note that the LDOS intensity and influential territory around individual vacancy-defects are much higher than that of vacancy chains. This observation explains the different average LDOS in these crystals. Although the two surfaces of the crystals have a similar number of Eu vacancies, their segregation around the antiphase boundaries in ‘crystal b’ largely decreases the amount of individual vacancies on the surface. The relatively large amount of random individual vacancies in ‘crystal a’ largely elevates the density of states.


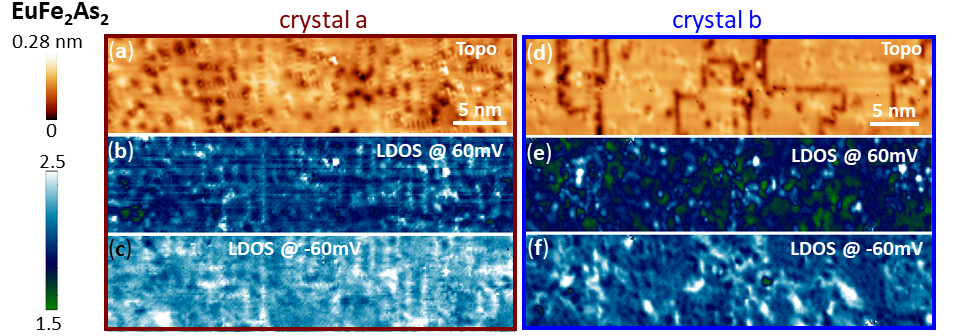


**Fig. S2**: EuFe_2_As_2_ local structures. (a, b, c) Topographic image, LDOS at 60 mV and -60 mV of √2 × √2 reconstructed surface on ‘crystals a’ (-60 mV, 800 pA). (d, e, f) Topographic image, LDOS at 60 mV and -60 mV of √2 × √2 reconstructed surface on ‘crystal b’ (-60 mV, 800 pA).

For both EuFe_2_As_2_ single crystals, high-pressure electrical-resistance measurements were performed using a diamond anvil cell (**Fig. S3a**), similar to earlier reports [2-4]. In situ, one of the anvils was symmetrically arrange and deposited with eight tungsten microprobes encapsulated in a homoepitaxial diamond film and are exposed only near the tip of the diamond to make contact with the EuFe_2_As sample at high pressure. The designer diamond anvil was beveled with flat diameter of 100 μm and culet diameter of ~ 300 μm. The distance between two opposite leads was ∼ 50 μm. The gasket was made from a 240 μm thick hardened spring steel foil pre-indented to 80 μm with a 120 μm diameter hole electrospark drilled through the center of the preindented region. Apart from eight electrical leads, two leads were used to set constant current through the sample and the two additional leads were used to monitor the voltage across the sample. A steatite pressure medium was employed in the electrical resistance measurements to assure electrical insulation of the sample from metallic gasket. The pressure was monitored by the ruby fluorescence technique and care was taken to carefully calibrate the ruby R_1_ and R_2_ emission in both samples at low temperatures. Although there are reports of the application of pressure on EuFe_2_As_2_ causing suppression of *T*_N_ and superconductivity onset at pressure values of ~ 2 to 3 GPa [5,6], here we want to explore the differences of pressure effects on the two crystals with different *T*_N_. A steatite pressure medium was employed in the electrical resistance measurements to assure some pressure uniformity. The temperature dependence of resistivity results is shown in **Fig. S3e**, up to ~ 20 GPa. For these crystals, clear anomalies due to Fe and Eu ordering are seen in the data. Although the feature due to Eu ordering is not changed for either crystals up to ~ 4 GPa, Fe ordering is greatly sensitive to pressure and the rate of *T*_N_ suppression for both crystals is similar. For ‘crystal b’ with smaller *T*_N_= 175 K, the drop in resistivity is noticed at lower pressure of 2.5 GPa, compared to ‘crystal a,’ with *T*_c_ setting in at 3.2 GPa. The highest *T*_c_ value for ‘crystal a’ is 36 K, and for ‘crystal b’ is 41 K. EuFe_2_As_2_ with sharper but lower *T*_N_ (‘crystal b’) gives a slightly higher superconducting dome, summarized in *T*-P phase diagram in **Fig. S4**.

**
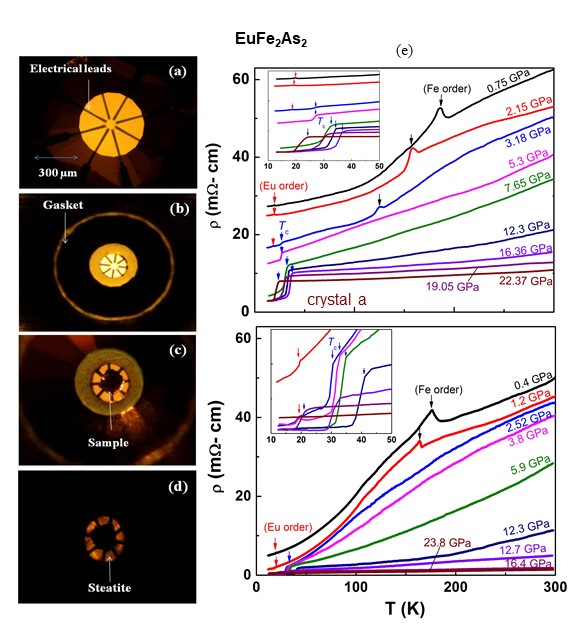
**

**Fig. S3.** (a) Eight probe designer diamond anvil used in high-pressure resistivity measurements of EuFe_2_As_2_ single crystal. The current and voltage contacts in the four probe electrical measurements are indicated. (a) The inset is the close-up of the diamond culet showing the eight shiny tungsten metal probes emerging near the center to make contact with the sample at high pressures. The metal probes are embedded in a chemical vapor deposited diamond layer elsewhere except for the contacts indicated. (b) Metallic gasket mounting in the designer diamond, (c) and (d) EuFe_2_As_2_ crystal loaded with a steatite pressure medium, thereby electrically insulating the sample from the gasket. (e) Temperature and pressure dependence of the electrical resistivity of EuFe_2_As_2_ ‘crystal a’ (top) and ‘crystal b’ (bottom).


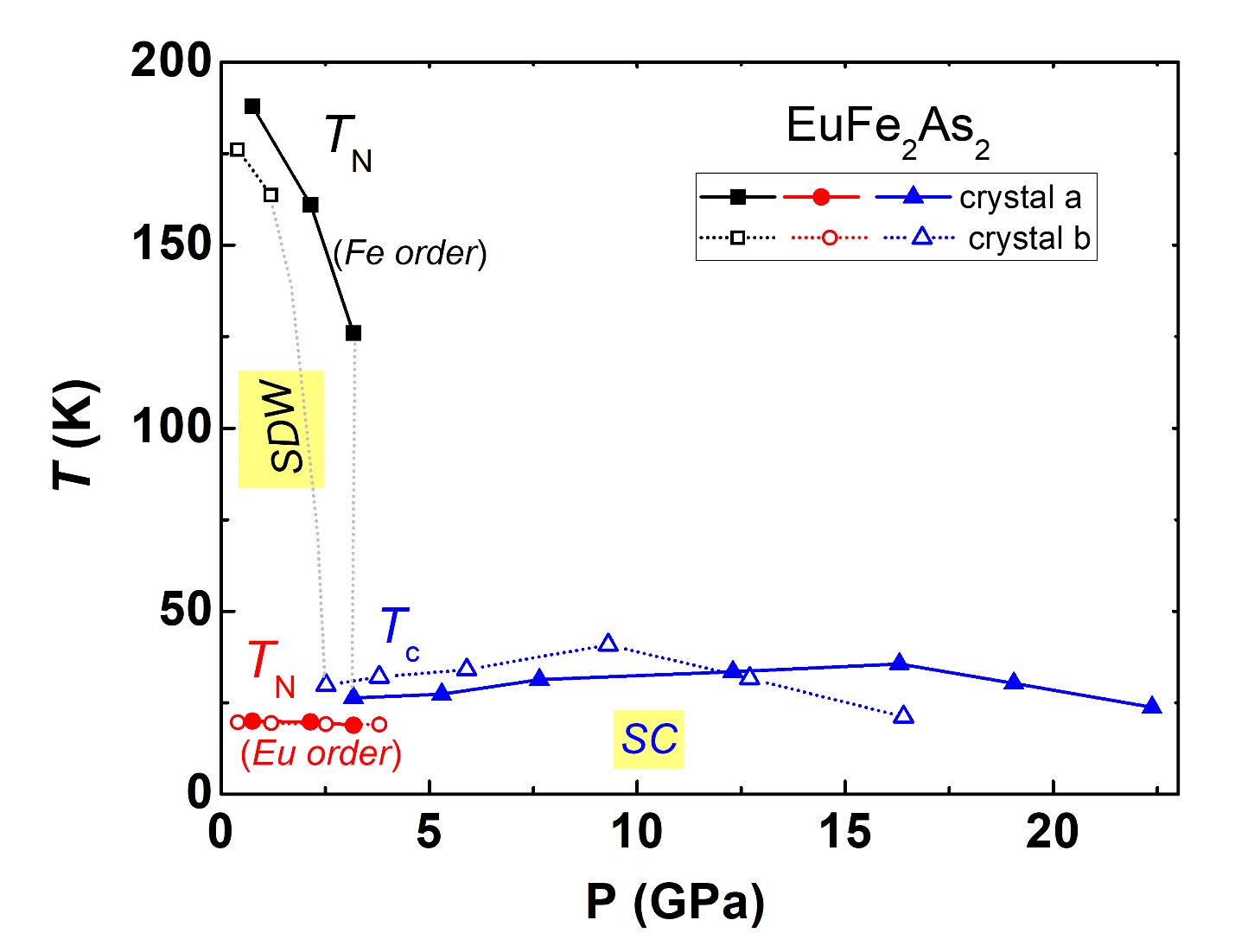


**Fig. S4**: Temperature-pressure phase diagram for the two EuFe_2_As_2_ crystals with different *T*_N_ at ambient pressure.

**References**

[1] Y. Xiao, Y. Su, M. Meven, R. Mittal, C. M. N. Kumar, T. Chatterji, S. Price, J. Persson, N. Kumar, S. K. Dhar, A. Thamizhavel, and Th. Brueckel, Phys. Rev. B **80**, 174424 (2009).

[2] S. T. Weir, J. Akella C. A. Ruddle, Y. K. Vohra and S. A. Catledge, Appl. Phys. Lett. **77**l, 3400 (2000).

[3] Y. K. Vohra and S. T. Weir, High pressure phenomenon Proc. Int. School of Physics—Enrico Fermi, Course XXLVII, Ed. R. J. Hemley, G. L. Chiarotti, M. Bernasconi, L. Ulivi (Bologna: IOS Press), p 87 (2002).

[4] G. Tsoi, A. Stemshorn, Y. K. Vohra, P. M. Wu, F. C. Hsu, Y. L. Huang, M. K. Wu, K. W. Yeh, and S. T. Weir, J. Phys: Condens Matter. **21**, 232201(2009).

[5] C. F. Miclea, M. Nicklas, H. S. Jeevan, D. Kasinathan, Z. Hossain, H. Rosner, P. Gegenwart, C. Geibel, and F. Steglich, Phys. Rev. B **79**, 212509 (2009).

[6] T. Terashima, M. Kimata, H. Satsukawa, A. Harada, K. Hazama, S. Uji, H. S. Suzuki, T. Matsumoto, and K. Murata, J. Phys. Soc. Jpn. **78**, 083701 (2009).
